# Frequency-specific electrogastrography as a non-invasive tool to measure gastrointestinal maturity in preterm infants

**DOI:** 10.1038/s41598-022-24110-y

**Published:** 2022-12-01

**Authors:** Akhil Chaudhari, Xinlong Wang, Lindsay Roblyer, Rinarani Sanghavi, Hanli Liu, Eric B. Ortigoza

**Affiliations:** 1 Department of Bioengineering, University of Texas at Arlington, Arlington, TX USA; 2 Division of Neonatal-Perinatal Medicine, Department of Pediatrics, UT Southwestern Medical Center, 5323 Harry Hines Blvd, Dallas, TX 75390 USA; 3 Division of Pediatric Gastroenterology, Department of Pediatrics, UT Southwestern Medical Center, Dallas, TX USA

**Keywords:** Paediatric research, Gastrointestinal system, Biomedical engineering

## Abstract

Enteral feeding is challenging in preterm infants because of gastrointestinal (GI) immaturity. Electrogastrography (EGG) is a non-invasive technology that measures gastric myoelectrical activity and can be utilized to measure changes that occur with maturation at different gestational ages (GA). Three gastric rhythms (GR) exist between 0.5–9 cycles per minute (cpm), namely, bradygastria (0.5 ≤ GR < 2 cpm), normogastria (2 ≤ GR < 4 cpm), and tachygastria (4 ≤ GR < 9 cpm). We aimed to characterize EGG-derived parameters for different GA by quantifying (1) power spectral density (PSD) and its spectral means at three GR bands (i.e., *m*PSD_*GR*_) and (2) the percent (%) time spent in each band. Data analyzed was from a longitudinal cohort of preterm infants (*n* = 51) born at early, mid, and term GA of < 29, 29–33, and ≥ 37 weeks, respectively. Weekly EGG monitoring was performed until 40 weeks’ postmenstrual age or discharge. Pre-, during, and post-feed data were analyzed for *m*PSD_*GR*_ at each GR band. Also, % bradygastria, % normogastria, and % tachygastria were calculated by continuous wavelet transform analysis. Results showed (1) *m*PSD values in normogastria and tachygastria during feeding increased with advancing GA, and (2) % normogastria increased with advancing GA regardless of GR ranges, suggesting EGG may measure GI maturity in preterm infants.

## Introduction

**Figure 1:**
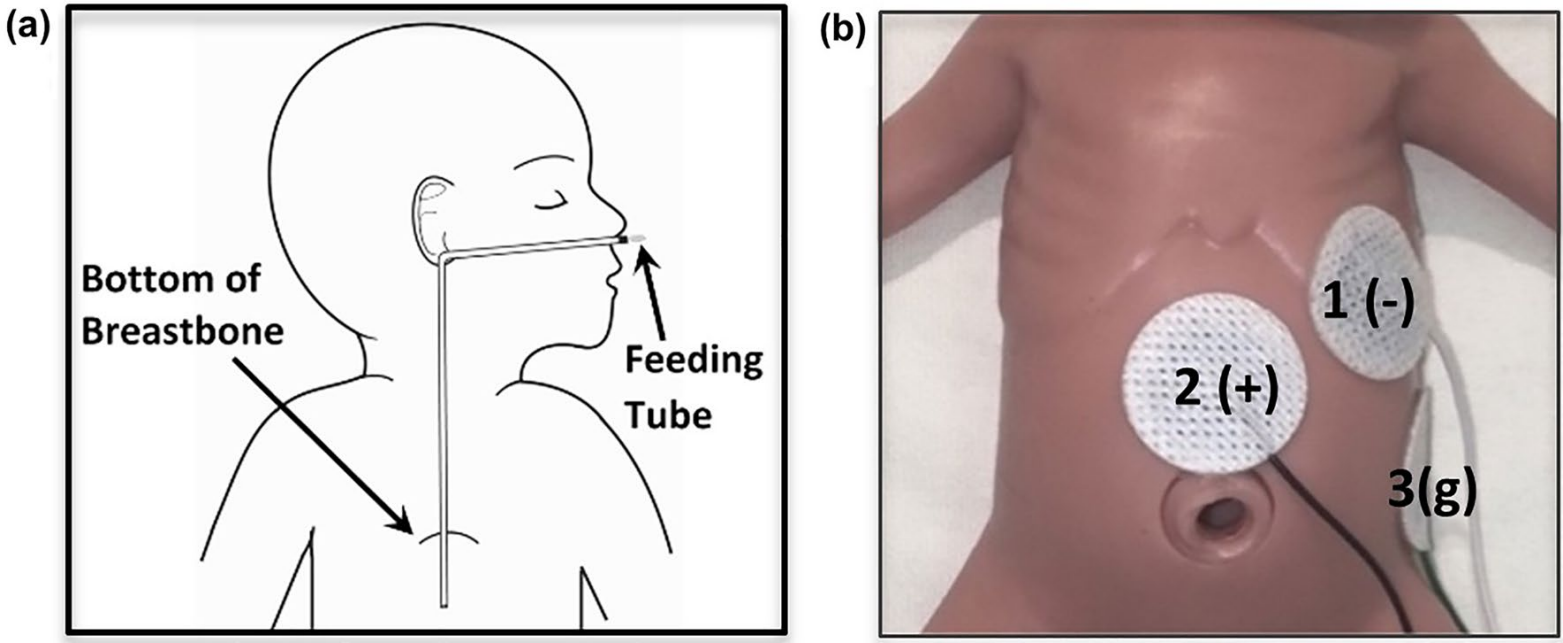
EGG Setup. (**a**) This cartoon shows a standard enteral feeding tube setup utilized in neonatal intensive care units. (**b**) Setup of three EGG electrodes. The negative electrode (marked by ‘1 (−)’) is placed on the left upper quadrant, near the mid-clavicular line. The positive electrode (marked by ‘2 (+)’) is placed midway between the bottom of breastbone and the belly button, slightly below the level of the negative electrode. The ground electrode (marked by ‘3(g)’) is placed at the mid-axillary line, below the left costal margin.

One of the most challenging tasks in neonatal intensive care is feeding preterm infants via the enteral route (Fig. 1a). Because of gastrointestinal (GI) immaturity, preterm infants can develop feeding intolerance, which leads to significant health problems including malnutrition and poor neurodevelopmental outcomes^1–3^. If a non-invasive, sensing methodology is available to measure GI maturity effectively in preterm infants, pediatric clinicians may be able to predict whether a preterm infant will tolerate feeds or develop intolerance. However, current clinical methods to sense feeding intolerance depend primarily on direct observations with non-specific measures, such as abdominal circumference, gastric residual volume, and presence of vomiting^4^. These measures are poor biomarkers to predict GI maturity and success of enteral feeding. Thus, it is highly desirable and necessary to develop or adapt a non-invasive device and methodology to quantify GI maturity for more accurate assessment of feeding readiness for preterm babies.

Electrogastrography (EGG) is a noninvasive technique used to record gastric myoelectrical activity using cutaneous electrodes placed on the abdominal skin over the stomach. Gastric myoelectrical activity consists of slow waves and spike potentials. EGG measures gastric slow waves or gastric rhythms (GR), which determine the frequency and propagation of stomach smooth muscle contractions^5^. While EGG records time series, the frequency-domain analysis is often used exhibiting three major frequency bands for three gastric rhythms between 0.5–9 cycles per minute (cpm), namely, bradygastria (0.5 ≤ GR < 2 cpm), normogastria (2 ≤ GR < 4 cpm), and tachygastria (4 ≤ GR < 9 cpm). Accordingly, spectral-power-related parameters are widely used to characterize the development or health conditions of the GI tract, such as dominant frequency, dominant power, power ratio, % bradygastria, % normogastria, % tachygastria, instability coefficient^6,7^.

EGG has been performed extensively in the adult population in the past 3 decades to investigate gastric dysfunctions, such as gastroparesis^8,9^, gastroesophageal reflux^10,11^, delayed gastric emptying (GE), abnormalities of central nervous system^12,13^ and functional dyspepsia^14,15^. A lower % normogastria was demonstrated in subjects with delayed GE than normal subjects^16^. A higher % tachygastria and reduced post-feed amplitude were observed in children with functional dyspepsia^17,18^. Effects of feeding composition and prokinetic agents on gastric motility have been studied extensively using EGG^19,20^. However, most of these studies focused on adults or older children, rather than preterm infants.

Because of its non-invasive nature, EGG has been used to monitor preterm infants with a setup as shown in Fig. 1b on a model infant. However, the use of EGG to characterize GI maturity in preterm babies during or post-feeding is sparse with dispersed results in the literature. Lange et. al. showed no significant difference in EGG dominant frequency between pre- and post-feeding and in the EGG power ratio between post-feeding power vs. pre-feeding power^7^. Precioso et. al. reported no significant difference in % normogastria between term and preterm neonates in the pre- and post-feed periods^15^. Ortigoza et. al. reported a significant increase in post-feed dominant power across the gastric spectrum (i.e., 0.5–9 cpm)^21^. Moreover, most of the studies were limited to the comparisons between the pre- and post-feeding periods, not *during* the actual feeding period. Most reported and analyzed parameters focused on dominant frequency, dominant power, power ratio, % bradygastria, % normogastria, % tachygastria, and instability coefficient. All these parameters were introduced several decades ago and may lack updated development on its mathematical and computational algorithms, which may partially account for the dispersed results in the literature.

Frequency-domain analyses used for EGG temporal data processing are different from power spectral density (PSD) analysis. The latter is a well-accepted signal-processing tool, utilized for decades in the engineering field and in the field of electroencephalogram (EEG) for analyses of multi-channel EEG time series. Power spectral density quantifies the distribution of rhythm power over frequency components of the signal and facilitates essential analyses of time series in many engineering and biomedical applications.

Thus, one novel goal of this study was to quantify and characterize PSD of EGG in preterm infants at three respective GR bands (i.e., *m*PSD_*GR*_) during three sub-feeding periods (pre-, during, and post-feed). The specific hypotheses in this study were that (i) *m*PSD increases during enteral feeding regardless of the GR frequency range, (ii) *m*PSD in the regular gastric rhythm (i.e., *m*PSD_*normo*_) increases during feeding with increasing gestational age, and (iii) percent normogastria (2 ≤ GR < 4 cpm) increases linearly with increasing gestational age for preterm infants. The overall goal was to develop and update the time–frequency analysis methodology to characterize EGG data and identify non-invasive biomarkers for sensing GI maturity in preterm infants at all 3 sub-feeding periods (pre-, during, and post-feed) instead of only pre-and post-feed as customary.

## Results

### Study cohort

Sixty-six participants were enrolled in the study after parental consent from 2017–2020. Fifteen participants were excluded (1 was too unstable for EGG recording, 1 had poor skin integrity, 3 had missing or poor data quality, and 10 did not have identical experimental setup because of missing near-infrared spectroscopy (NIRS) measurements)). Five subjects withdrew prior to completion of the protocol because of reasons unrelated to the study. For all withdrawn subjects, parental permission was obtained to analyze and include any data that was acquired prior to the date of withdrawal. Thus, 51 neonates (47 preterm, 4 term) were included in the study (See Supplementary Table S1 for participant demographics). Subjects were stratified into 3 GA groups: early (< 29 weeks), mid (29–33 weeks), and term (≥ 37 weeks). None of the subjects developed any study-related skin complications.

### Quantifications of *m*PSD at three gastric frequency bands

**Figure 2:**
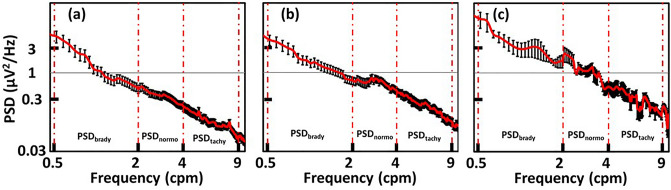
Power spectral density (PSD) plots from different gestational age groups. An example of group-averaged PSD plots taken from (**a**) early GA (*n* = 25), (**b**) mid-GA (*n* = 22), and (**c**) term infants (*n* = 4) in the During-feed period averaged between Feeding 1 and Feeding 2. X-axis is the GR frequency of EGG in cpm, and y-axis is the power spectral density in μV^2^/Hz. The red vertical lines mark the GR frequency bands of bradygastria (brady), normogastria (normo), and tachygastria (tachy). The error bars represent the standard error of the mean from four term infants.

After following the four steps given in Methods Section “EGG power spectral density (PSD) and spectral means at three gastric frequency bands”, we were able to obtain group-level PSD of EGG in the GR frequency range of 0.5–9 cpm during different feeding periods. Figure 2 shows PSD plots from (a) early GA (*n* = 25), (b) mid GA (*n* = 22), and (c) term (*n* = 4) infants in the During-feed period averaged between Feeding 1 and Feeding 2. By taking the spectral average across three GR bands (as marked in Fig. 2), we obtained *m*PSD_brady_, *m*PSD_normo_, and *m*PSD_tachy_, respectively, for each GA infant group and then for each of the three sub-feeding periods (i.e., pre-, during, and post-feed).

### Gestational age-dependent *m*PSD_GR_ values during the feeding periods

**Figure 3:**
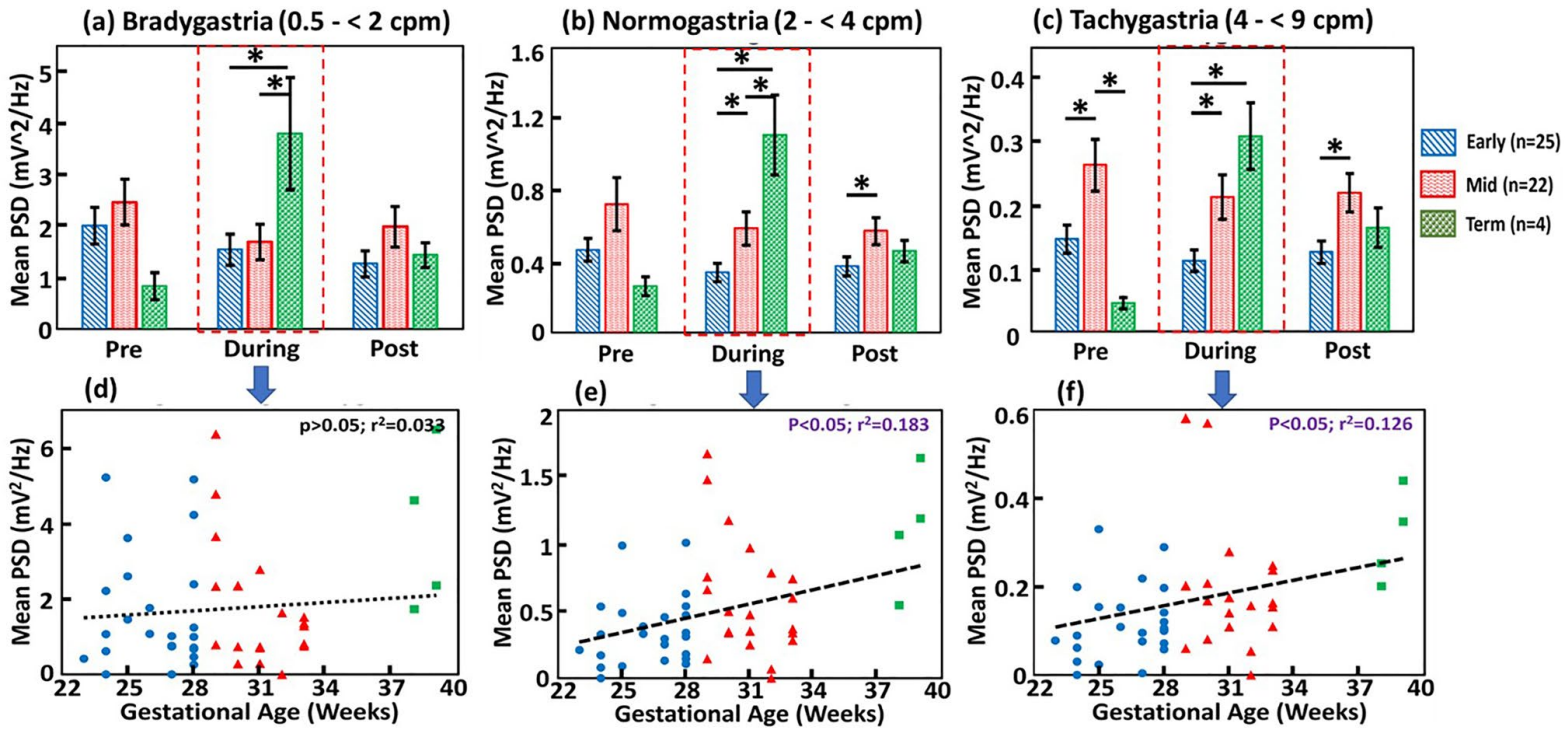
Mean power spectral density (*m*PSD) increases with maturation during feeding. Group-level comparisons of GA-dependent *m*PSD values (mV^2^/Hz) taken pre-, during, and post-feeding period in (**a**) bradygastria, (**b**) normogastria, and (**c**) tachygastria, respectively. “*” indicates the statistical significance with *p* < 0.05; the red boxes mark the During-feed periods. (**d**–**f**) show the corresponding linear regressions of (**a**) to (**c**) between GA of infants and *m*PSD, respectively, in the During-feed periods.

Figures 3(a,b,c) show among-group comparisons of GA-dependent *m*PSD values at all three GR (bradygastria, normogastria, and tachygastria) bands, respectively. The most consistent and noticeable feature among these three panels is the significant increase in *m*PSD values in the During-feed period as the GA of infants increased. Corresponding p-values for different pairs of comparisons are listed in Supplementary Table S2.

To better understand this feature, Fig. 3(d,e,f) plots a linear regression between the GA of the infants versus *m*PSD during feeding in each GR case, revealing that the *m*PSD_normo_ and *m*PSD_tachy_ values during feeding are significantly higher for more mature preterm infants (mid) than less mature (early). We acknowledged that the sample size for term babies was limited; however, these served as a reference to demonstrate the overall trend for comparison. Furthermore, during the Post-feed period, significant increases in *m*PSD_normo_ (*p* = 0.02) and in *m*PSD_tachy_ (*p* = 0.006) were observed between the early and mid GA infants (see Supplementary Table S2).

### No alteration in *m*PSD_GR_ in preterm neonates across three feeding periods and three GR bands

**Figure 4:**
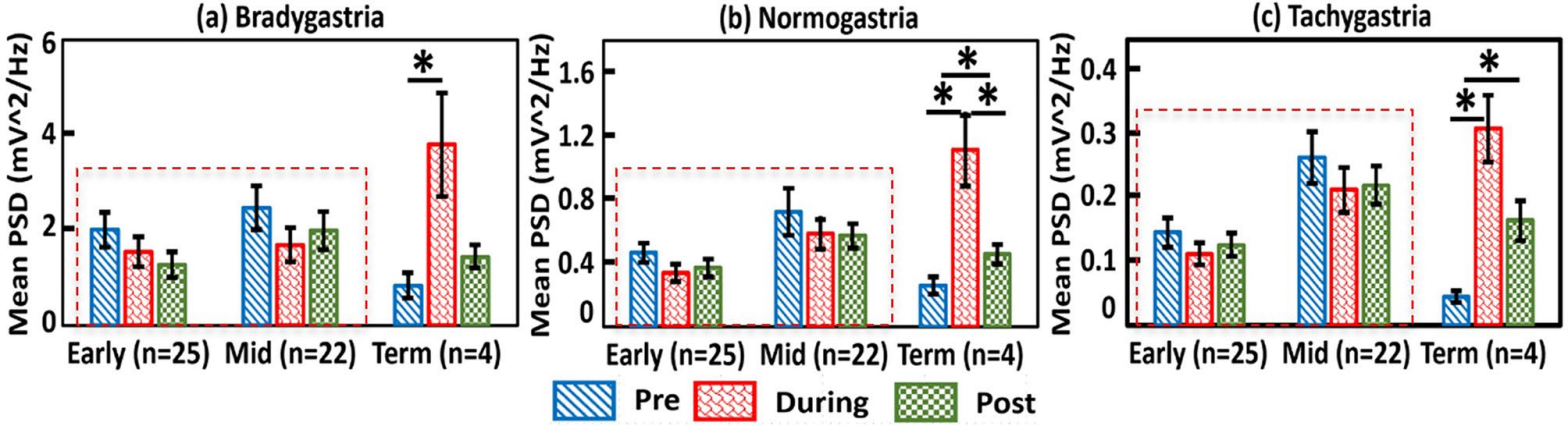
Enteral feeding did not influence the mean power spectral density (mPSD) in preterm infants. Group-level comparisons within each of the three GA groups of the *m*PSD values across the three feeding periods in (**a**) bradygastria, (**b**) normogastria, and (**c**) tachygastria, respectively. In each panel, the y-axis denotes the *m*PSD, and the x-axis represents the neonate GA groups. The red dashed boxes outline no significant difference in *m*PSD across the three feeding phases in both early GA and mid GA infants at all three GR bands. A significant increase in *m*PSD is marked by “*” and determined by *p* < 0.05. Error bars denote the Standard Error of the Mean for the respective *m*PSD values across three GA groups and in each GR band.

We examined within-group differences in *m*PSD across the three feeding periods for each of the GR bands in each GA group, as shown in Fig. 4(a,b,c), respectively. One-way ANOVA results demonstrated no significant difference across the three sub-feeding periods in both early GA and mid GA infants (marked by the red dashed boxes in the three panels of Fig. 4) at all three GR bands. ANOVA followed by Tukey’s post-hoc test revealed that *m*PSD values of term babies had highly significant differences between During-feed versus Pre-feed and in all three GR bands (bradygastria with *p* = 0.03; normogastria with *p* = 0.01; tachygastria with *p* = 0.002). Furthermore, for the term infants, the group-level values of *m*PSD_normo_ altered significantly between Pre-feed vs. Post-feed (*p* = 0.04) and between During-feed and Post-feed (*p* = 0.03); similarly, the values of *m*PSD_tachy_ altered significantly between Pre-feed versus Post-feed (*p* = 0.01). Even though term babies were limited in number (*n* = 4), the overall inability of preterm infants to alter EGG PSD across all three GR bands would still be a qualitative reflection of gastric immaturity because the term infants showed significant increases in PSD regardless of GR band during the feeding period.

### Increases in percent time in normogastria with increasing GA in all three sub-feeding periods

**Figure 5:**
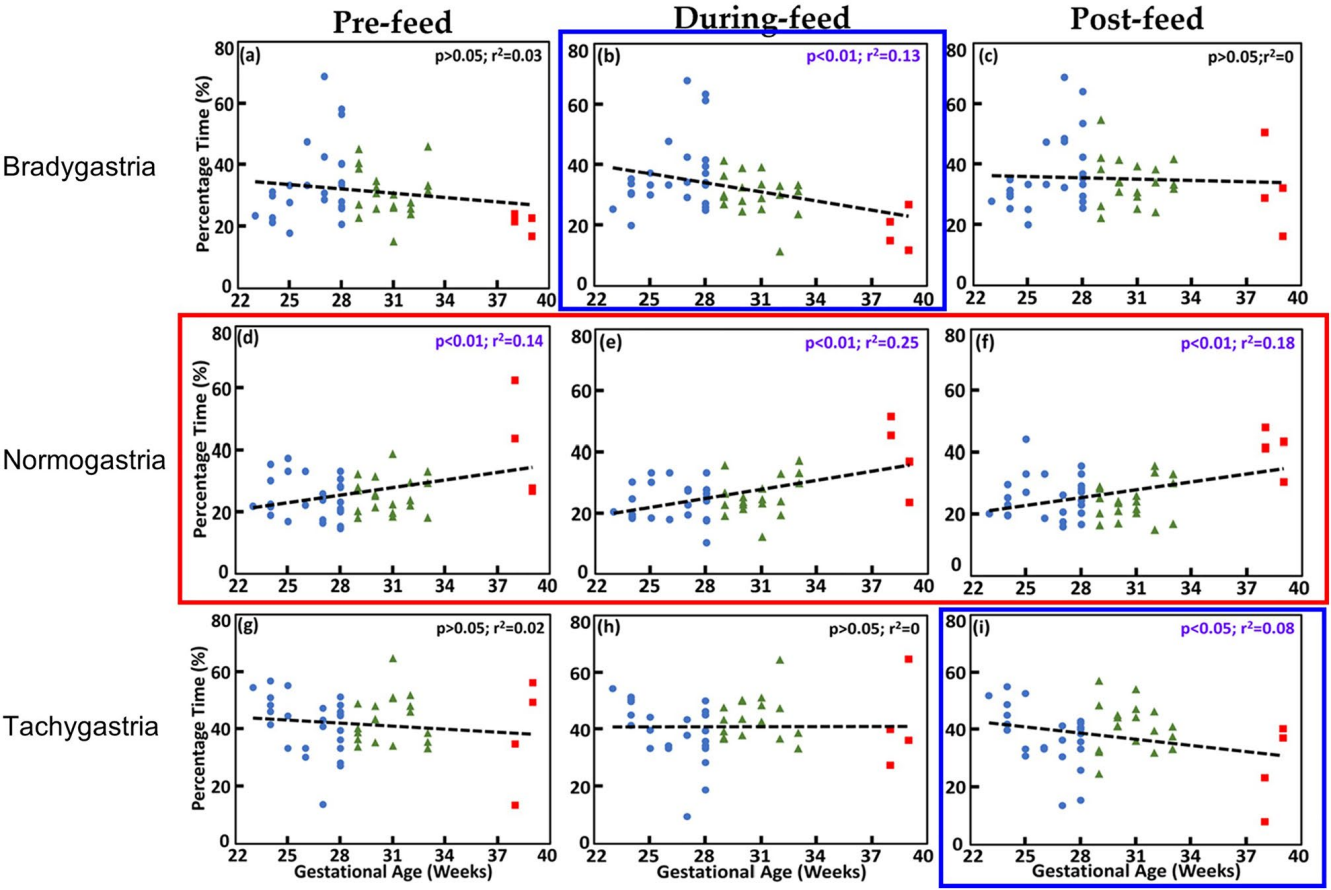
Percentage time spent in each gastric rhythm (GR) band. Percentage time spent in each GR band, namely, % bradygastria (top row), % normogastria (mid row), and % tachygastria (bottom row) for all the three sub-feeding periods. Panels (**a**–**c**) on the top row depict the % bradygastria in the 3 sub-feeding periods (pre-feed, During-feed, and post-feed, respectively). Panels (**d**–**f**) on the middle row show the % normogastria in the respective three sub-feeding periods. Panels (**g**–**i**) on the bottom row illustrate the % tachygastria during the respective three sub-feeding periods. A positive correlation was observed between the % normogastria and GA during each of the three sub-feeding periods, as outlined by the red box. A negative correlation was demonstrated between % bradygastria and GA in the During-feed period and between % tachygastria and GA in the Post-feed period (blue boxes).

After the non-stationary EGG signal was processed by continuous wavelet transform (CWT), the percentage time spent in each GR band was as described in the 7 steps in Methods Section “Percentage of time spent in each gastric rhythm during each sub-feeding period” for each GA group during three feeding periods and in 3 separate GR bands. Figure 5 demonstrates the relationship between percentage time (in each GR) versus GA for each of the three sub-feeding periods (along rows) in each of the three GR bands (along columns). Linear regression analysis showed a positive and significant linear correlation between % normogastria and GA in the Pre-feed (*p* < 0.01, ${r}^{2}$=0.14), During-feed (*p* < 0.01, ${r}^{2}$=0.25), and Post-feed (*p* < 0.01, ${r}^{2}$=0.18) periods, as outlined by the red box in the middle row. In addition, negative correlations between % bradygastria versus GA During-feed (*p* < 0.01, ${r}^{2}$=0.13) and between % tachygastria and GA Post-feed (*p* < 0.05, ${r}^{2}$=0.08) were observed, as indicated by the blue boxes in Fig. 5. Feeding type (maternal own milk, donor human milk, or formula), addition of human milk fortifier, Calories, and volume were not different between GA groups (see Supplementary Table S3).

## Discussion

Electrogastrography is a non-invasive, practical, and safe method to measure gastric myoelectrical activity in neonates. It is highly desirable that EGG could facilitate development of characteristic biomarkers/features to identify and estimate GI maturity in preterm infants. The overall goal was to develop and update the time–frequency analysis methodology to characterize EGG data and identify non-invasive biomarkers for sensing GI maturity in preterm infants at all 3 sub-feeding periods (pre-, during, and post-feed) instead of only pre-and post-feed as customary. Similarities and differences of this study compared with prior studies are listed in Supplementary Table S4. We hypothesized in this study that (i) spectral *m*PSD increases during enteral feeding regardless of the GR frequency range, (ii) *m*PSD in the regular gastric rhythm (i.e., *m*PSD_*normo*_) increases during feeding with increasing gestational age, and (iii) percent normogastria (2 ≤ GR < 4 cpm) increases linearly with increasing gestational age for preterm infants. To support our hypothesis, we conducted the study from a longitudinal cohort of preterm infants (*n* = 51) born at early, mid, and term GA of < 29, 29–33, and ≥ 37 weeks, respectively. After careful and thorough data analyses, we obtained several key findings, as discussed in more detail as follows.

We facilitated a novel and thorough approach to perform EGG time–frequency analysis of a longitudinal cohort of 51 neonates across three gastric rhythms, namely, bradygastria, normogastria, tachygastria and across three sub-feeding periods. Specifically, we quantified spectral means of EGG power spectral densities at the three GR bands for preterm infants and found distinct features that were linearly associated with gastric immaturity. In this way, we characterized novel and frequency-specific GR features of EGG, namely, *m*PSD_brady,_ *m*PSD_normo,_ and *m*PSD_tachy_. Second, we utilized and implemented CWT for determination of percentage time of non-stationary EGG rhythms spent in the three GR frequencies across three sub-feeding periods (i.e., pre-feed, during-feed, and post-feed) for preterm and term neonates. The advantage of utilizing CWT was the ability to analyze changes in gastric myoelectrical activity that occurred because of enteral feeding and the changes that occurred with maturation.

We demonstrated that *m*PSD values during the feeding period increased significantly in all three GR bands with increases of gestational age (Fig. 3a,b,c)). A significant and positive linear relationship between the *m*PSD_normo_ and GA at the normal gastric rhythm (Fig. 3e) during feeding is expected because it suggests developmental process and functional growth of the GI system of preterm babies. Also, changes in *m*PSD_tachy_ (Fig. 3f) were linearly in proportion to GA. We interpreted this observation as follows: because of GI immaturity in very preterm infants, the strength of the myoelectrical signal is low; however, it increases with increasing GA and GI maturity. Thus, the increase in *m*PSD_tachy_ with GA reflects this increase in signal strength with maturity. This is not to be confused with the percent time spent in tachygastria (% tachygastria), which is a different parameter that will be discussed later in this section. Because PSD is signal-amplitude dependent, low amplitudes of gastric waves in preterm infants were automatically quantified. Both *m*PSD_normo_ and *m*PSD_tachy_ are absolute values and thus can be comparable among different infants, they may have great potential to be developed as biomarkers for assessing the GI maturity of preterm neonates.

We demonstrated that preterm infants at the GA of < 34 weeks were unable to alter/change their *m*PSD at any of the three GR frequencies across the three sub-feeding periods (Fig. 4). The absence of change in gastric myoelectrical activity in response to feeding suggests GI immaturity.

To-date, the comprehensive relationship between *m*PSD values versus three feeding periods (i.e., pre-, during, and post-feeding) at three GR bands has not been established until the present study, particularly for preterm infants. EGG has been utilized to study myoelectric changes during postnatal maturation^17,22–26^. In older children and adults, EGG dominant power is expected to increase in response to feeding^27–30^. These observations can be interpreted as follows: With an increase in GA, gut muscles during the period of feeding and post-feeding operate more and thus exert more myoelectrical power than during pre-feed, indicating a normal developmental process. The absence of change in the *m*PSD of preterm infants in all 3 sub-feeding periods regardless of GR frequency band suggests immature or dysfunctional myoelectrical activity. The potential effect of feed type and fortification on the myoelectrical signal was outside the scope of this study; however, this needs to be evaluated in future studies with larger sample sizes.

In this study, we have implemented a unique and novel method for quantifying the % time spent in each GR frequency band during each sub-feeding period using continuous wavelet transform, CWT. Previous literature has suggested the use of running spectral analysis methods, such as short-term Fourier transform (FT) analysis^15,31–34^. CWT is useful in analyzing the non-stationary signal and also allows to filter out stationary and non-stationary signals^35,36^. Because the EGG measurement is obtained from preterm babies longitudinally, the signal is expected to be highly non-stationary, and thus CWT is a more appropriate means used for time–frequency analysis. In the field of EGG^37–44^ this is the first research report to utilize and implement CWT for determination of percentage time of non-stationary EGG rhythms spent in different gastric rhythm frequencies across three sub-feeding periods.

We demonstrated a positive correlation between % normogastria and GA during all 3 sub-feeding periods (Fig. 5). This positive correlation between % normogastria and GA might suggest normal gastric maturity with increase in gestational age. Furthermore, our results demonstrated that % bradygastria during feed and % tachygastria post-feed had a negative correlation with GA. Children with functional dyspepsia have been reported to have a higher % tachygastria when compared to normal healthy children^17,18^. An increase in % tachygastria may suggest delayed gastric emptying^34^. A decrease in % tachygastria with increasing GA suggests a developmental process and a decrease in gastric dysrhythmias with maturation. To-date, this is the first thorough investigation in preterm infants to report linear and significant changes between the increasing GA and in percentage time spent in different GR during all three sub-feeding periods (inclusive of during feed). Moreover, because of the linear and significant correlation between GA and the % normogastria, the latter (as denoted by %T_normo_) is also possible to be further developed as a biomarker because %T_normo_ is independent of sub-feeding period. In this study, the non-linear, harmonic components of low and normal slow waves (i.e., bradygastria and normogastria) were not included in the time–frequency spectrogram after CWT of EGG time series. This is because CWT is a linear method^45^ that emphasizes only linear effects for the signals under analysis. However, it would be more mathematically rigorous and physiologically realistic if a non-linear analysis approach is taken to investigate EGG time series since gastric myoelectrical activity in humans and infants are highly complex and nonlinear. Such a topic is beyond the scope of this study and will be a future investigation.

We demonstrated that *m*PSD values during enteral feeding regardless of the GR frequency range increased significantly with increases in gestational age (Fig. 3), suggesting maturation of myoelectrical signal. Next, we reported that *m*PSD in the regular gastric rhythm (i.e., *m*PSD_*normo*_) increased linearly during feeding with increasing gestational age. Furthermore, preterm infants < 34 weeks’ GA were unable to alter/change their *m*PSD values across the three sub-feeding periods (Fig. 4), suggesting GI immaturity. Last, % normogastria increased with increasing GA regardless of sub-feeding period for preterm infants. All these observations supported and confirmed the hypotheses proposed in the beginning of the study. Based on the results we conclude that several EGG-derived parameters, such as *m*PSD_normo_, *m*PSD_tachy_, and %T_normo_, have potential to be further developed as tools to assess GI maturity and predict feeding readiness. However, further investigations need to be explored with a larger cohort of preterm and term infants.

This study had several limitations. First, the sample size for the term infants was small, preventing us from drawing more reliable conclusions for the term babies. As stated previously, term infants in this study were intended to serve more as a reference than a control group. Second, the time separations between two Feedings should be more clearly defined as the Post-feed for Feeding 1 and the Pre-feed for Feeding 2. More investigations with appropriate methods to classify Post-feed and Pre-feed are desirable. Third, patient body position in this observational study was at the discretion of the clinical team. Although all babies were in the supine position at the beginning of the EGG sessions, some infants required repositioning by the bedside nurse to improve vital signs. Because EGG is sensitive to motion artifacts, we encouraged minimal, unnecessary stimulation by the clinical staff. We did not monitor or delete motion artifacts before spectral analysis. We utilized Welch’s method, a popular analysis method for reduction of noise in the estimated PSD^46,47^. While Welch’s method enables to reduce EGG noises caused by motion artifacts, it would be more effective if a motion sensor, such as an electromyogram (EMG), can be implemented for detection of infants’ movement. Such EMG signal concurrently recorded with EGG will permit removal of motion artifacts accurately by a regression approach”. Other advanced algorithms for motion artifact identification, removal, and denoising are needed to achieve higher quality of EGG data. These may be necessary before EGG can serve as a non-invasive bedside tool to assess feeding readiness in preterm babies.

## Conclusion

Electrogastrography is a non-invasive bedside tool that has the potential to become a non-invasive means to assess GI maturity in preterm infants. In this study, we confirmed that (1) gastric myoelectrical activity of preterm infants had no alteration of its EGG power spectral density in response to feeding or in the post-feeding period, (2) *m*PSD values in normogastria (2 ≤ GR < 4 cpm) and tachygastria (4 ≤ GR < 9 cpm) increased linearly and significantly with increases of gestational age, and (3) % normogastria increased linearly with increasing gestational age regardless of sub-feeding period. All these quantified parameters have promising potential to serve as biomarkers for assessment of feeding readiness in preterm infants, and thus decreasing morbidity in this population. However, the findings in this study are relatively new with a limited sample size, particularly for term babies. Further clinical investigations with a larger sample size of both preterm and term babies are necessary for clinically meaningful conclusions.

## Methods

### Study design

The study design was a longitudinal, prospective cohort study from 2017–2020 in which participants underwent weekly EGG monitoring until 40 weeks postmenstrual age (PMA), discharge, or death, whichever came first.

### Neonates

Sixty-six infants (61 preterm and 5 term) from the neonatal intensive care unit (NICU) at Parkland Health and Hospital System and Children’s Health in Dallas, Texas, were enrolled from 2017–2020. Informed consent was obtained from all subjects and/or their legal guardian. The Institutional Review Board at UT Southwestern Medical Center approved the study. This study was performed in accordance with relevant guidelines and regulations.

Inclusion and exclusion criteria: Participants were preterm infants < 34 weeks’ gestational age (GA) and term infants ≥ 37 weeks’ GA at birth. Term infants were enrolled as a study reference. Infants were excluded if they had a known congenital or chromosomal disorder, significant clinical instability, or major skin abnormalities that would preclude placement of skin electrodes. Neonates were stratified by GA as early (< 29 weeks of GA), mid (29–33 weeks of GA), and term (≥ 37 weeks of GA).

Electrogastrography data from this pilot study was obtained from a longitudinal study of GI development^48^ to investigate coherent features between gastric myoelectrical activity (by EGG) versus abdominal oxygenation (by near infrared spectroscopy; NIRS). Infants without NIRS data were excluded from EGG analysis in this study to avoid any bias due to un-identical experimental setups. Thus, we included 51 (47 preterm and 4 term) infants, who had concurrent EGG and NIRS readings, for the data analysis in this study.

### Setup and experimental paradigm

#### Electrogastrography (EGG)

Neonatal EGG electrodes were placed on each neonate’s abdominal skin as validated and described in Chen et al. 1999 and others^21,49–51^. The setup was limited to 3 electrodes (see Fig. 1b) due to the abdominal size of neonates. Data acquisition was carried out using the data acquisition unit (BIOPAC® Systems, Inc., Goleta, CA) of the BIOPAC® MP36R System.

#### Feeding

Feeding was at the discretion of the clinical team. Because most babies in the NICU were fed every 3 h, the timing of EGG recording was 6 h to ensure that 2 pre-, 2 during, and 2 post-feed periods were included. EGG recordings included the 3 sub-feeding periods (pre-, during, and post-feed). The EGG data were further analyzed, presented, and described below.

### Data processing

#### EGG data preprocessing

Raw EGG data were collected at a sampling frequency of 2000 Hz and then preprocessed using MATLAB (Mathworks®, Natick, Massachusetts) as follows: (1) data were down sampled to 500 Hz using MATLAB function “downsample”; (2) a 3rd order polynomial fit was performed with MATLAB function “polyfit” for data detrending to obtain a temporal trend for the down-sampled time series; (3) the fitted trend was subtracted from the down-sampled data^52,53^; (4) the detrended time series was filtered using a low-pass filter at 1 Hz with the “filtfilt” filter in MATLAB to avoid any filtering-induced phase shift.

#### Selections of sub-feeding periods

**Figure 6:**
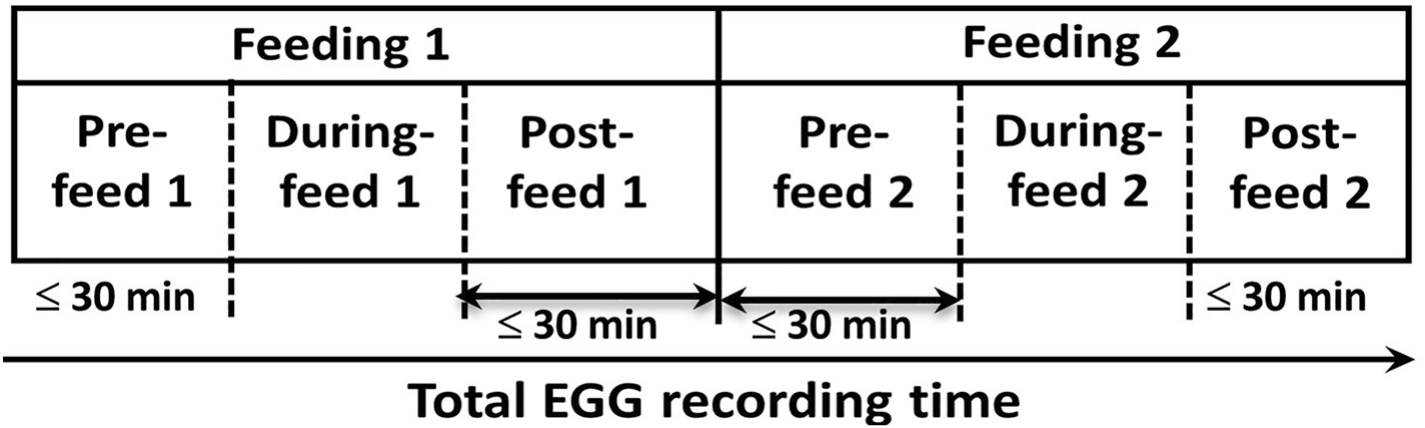
Feeding periods during EGG recording. EGG data were first sectioned into two feeding periods. Feeding 1 included Pre-feed 1, During-feed 1, and Post-feed 1. Feeding 2 included Pre-feed 2, During-feed 2, and Post-feed 2.

We divided the pre-processed data into pre-, during, and post-feed periods. Divisions of sub-feeding periods are demonstrated in Fig. 6. Each infant’s EGG recording included two major feeding periods, namely, Feeding 1 and Feeding 2, which were each further divided into pre-, during, and post-feed. During-feed 1 and During-feed 2 were extracted within Feeding 1 and Feeding 2, respectively, based on the actual duration of administration of the enteral feed. The time for pre-feed 1 was chosen to be 30 min. Similarly, the post-feed 2 would be 30 min long. The time interval between the end of During-feed 1 and the start of During-feed 2 was then divided into two equal-time halves. The first temporal half (30 min) was labeled as Post-feed 1; the second temporal half (30 min) was labeled Pre-feed 2, as illustrated schematically in Fig. 6. Pre-feeding and post-feeding periods that were shorter than 30 min were also included.

#### EGG power spectral density (PSD) and spectral means at three gastric frequency bands

There were several steps taken in this processing routine.

##### Step 1

The PSD across the entire gastric frequency band (0.5–9 cpm) during each of the six feeding periods (i.e., Pre-feed 1, During-feed 1, Post-feed 1, Pre-feed 2, During-feed 2, and Post-feed 2) was calculated using the native MATLAB function of “pwelch” (with the down-sampled frequency of 500 Hz, 4-min window and 2-min overlap) for each neonate. The 4-min window resulted in a frequency resolution of 0.004 Hz (or 4 mHz) in PSD calculation between 0 to 1 Hz. The purpose of applying Welch’s method was to minimize or reduce the noise caused by motion artifacts, imperfect, and finite data^46,47^.

##### Step 2

Mean PSD values over three sub-feeding periods were quantified by averaging them between Pre-feed 1 and Pre-feed 2, During-feed 1 and During-feed 2, and Post-feed 1 and Post-feed 2.

##### Step 3

Spectral means of PSD across each band were performed for all three gastric rhythm bands of interest, *m*PSD_GR_, where “GR” represents Bradygastria (0.5 ≤ *f* < 2 cpm; 0.008 ≤ *f* < 0.033 Hz), or Normogastria (2 ≤ *f* < 4 cpm; 0.033 ≤ *f* < 0.067 Hz), or Tachygastria (4 ≤ *f* < 9 cpm; 0.067 ≤ *f* < 0.15 Hz) during each sub-feeding period.

##### Step 4

Group-level values of *m*PSD_brady_, *m*PSD_normo_, and *m*PSD_tachy_ were calculated across three GA infants, namely, early GA (<29 weeks), mid GA (29–33 weeks), and term GA (≥37 weeks) infants for each of the three sub-feeding periods (i.e., pre-, during, and post-feed).

##### Step 5

Statistical analysis: the group-level *m*PSD_brady_, *m*PSD_normo_, and *m*PSD_tachy_ were compared among three sub-feeding periods for each of the three neonate groups at each GR band separately using one-way ANOVA, followed by Tukey’s post-hoc test. Also, linear regression was performed to determine the relationship between GA versus *m*PSD_GR_ at each GR frequency band for each GA group.

#### Continuous wavelet transform (CWT) for non-stationary EGG signal processing

**Figure 7:**
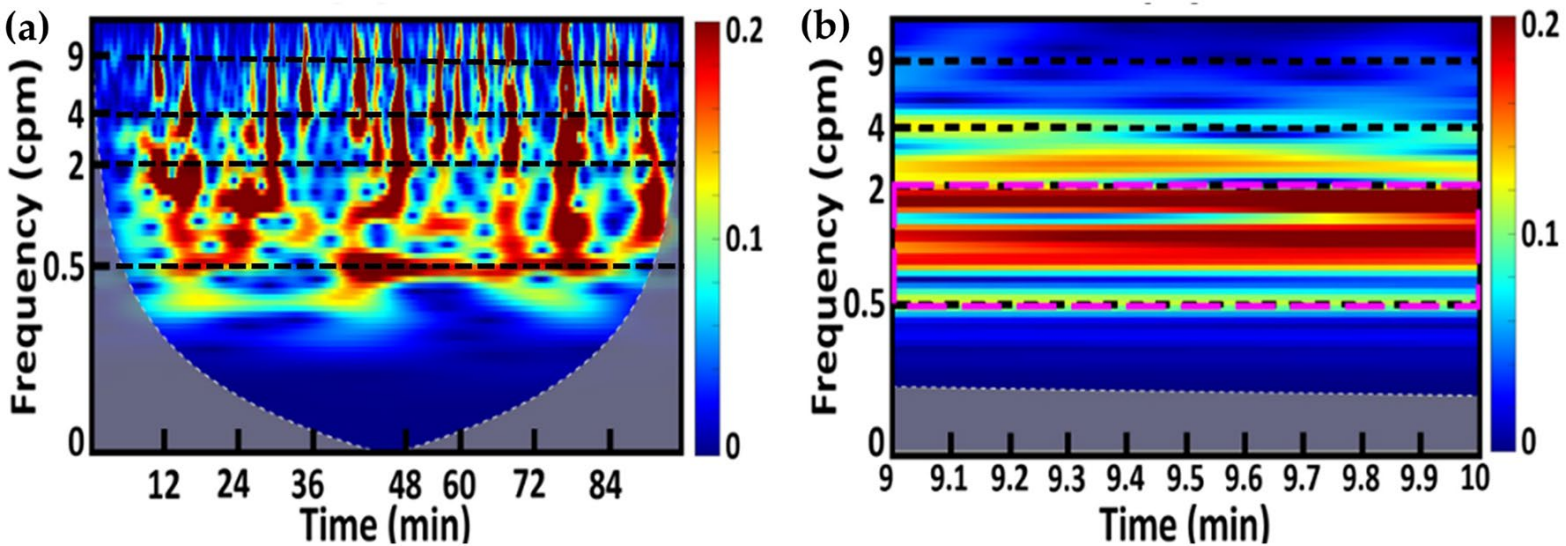
Continuous wavelet transform. (**a**) A CWT-derived spectrogram from a neonate during Feeding 1, including 30-min pre-feed, 30-min during feed, and 36-min post-feed. (Note that this 36-min Post-feed was used only for plot purpose. Only the 30-min Post-feed time was considered for both PSD and % time calculations). The horizontal x-axis represents time in min; the vertical y-axis denotes the frequency in cpm between 0 and 10 cpm plotted in log scale; the color bar represents wavelet transform power of an EGG time series. Several horizontal dashed lines mark the three GR bands of interest (bradygastria: 0.5 ≤ GR < 2 cpm; normogastria: 2 ≤ GR < 4 cpm; tachygastria: 4 ≤ GR < 9 cpm). (**b**) A zoomed spectrogram at 9–10 min from panel (**a**). The pink dashed box indicates the dominant frequency band for this particular minute being considered.

Because EGG signal is non-stationary, CWT would be a rigorous processing method for its time–frequency analysis to quantify its time-dependent spectral features. To perform CWT, we first filtered EGG data between 0.5 and 15 cpm using a zero-phase bandpass Butterworth filter in MATLAB. Next, each filtered EGG time series was analyzed to achieve a time–frequency spectrogram per each of the sub-feeding periods including both Feeding 1 and Feeding 2 (see Fig. 6) using a native MATLAB function “cwt” for each neonate. Figure 7a shows an example of CWT-derived spectrogram from one infant during Feeding 1 period (96 min), while Fig. 7b is a zoomed spectrogram within 9–10 min. Gray areas are called Cone of Inference (COI) that result from the edge effect of CWT^54^. Values within COI are considered highly non-reliable and thus excluded from the analysis.

#### Percentage of time spent in each gastric rhythm during each sub-feeding period

Another characteristic feature of EGG signal from preterm neonates was percentage time spent in each GR frequency band during three sub-feeding periods. Term babies are expected to have higher % normogastria, than early and mid GA preterm infants. Preterm infants (early and mid GA) are expected to have a higher % bradygastria, and % tachygastria, than term infants, suggesting GI immaturity. To determine the % of time that each GA infant group spent at each of the three GR bands, the following steps were executed:

##### Step 1

Three EGG GR frequency bands, as marked in Fig. 7a, were identified. Accordingly, spectral and temporal averaging over each minute of respective CWT power values for each GR band were performed.

##### Step 2

The dominant frequency band per minute among the three GR-specific band (i.e., bradygastria, normogastria, and tachygastria) was determined by choosing the largest ratio between each of the three GR-specific CWT powers versus the total power over the entire 0.5–9 cpm band at the respective time. As an example, the pink box in Fig. 7b outlines the GR band for bradygastria, which had the highest CWT power ratio and thus was considered the dominant frequency for the 9–10 min interval. In general, the ratios at the three GR bands reflected frequency distributions of gastric myoelectrical activity of the infant in that minute.

##### Step 3

**Figure 8:**
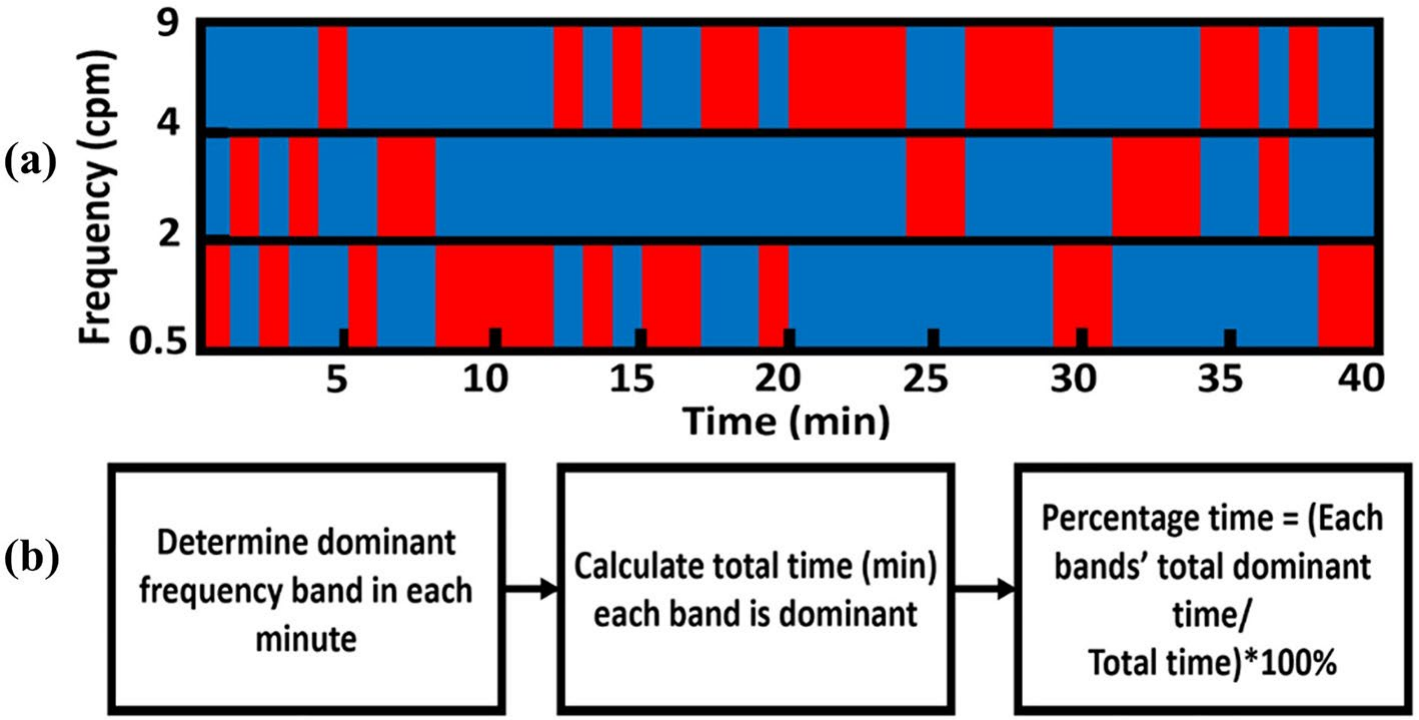
Dominant frequency band partition. (**a**) An example of minute-dependent dominant frequency band partition map across a time period (0–40 min). X-axis denotes the time in minutes; Y-axis represents three EGG rhythm frequency bands in cycles per minute (cpm). Red boxes indicate the dominant frequency band for each minute. (**b**) A flow chart of the procedure to calculate the % time spent in each GR band for each chosen time period.

The two steps described above were repeated to identify the dominant frequency band for each minute over each of the six sub-feeding periods (i.e., Pre-feed 1, During-feed 1, Post-feed 1, Pre-feed 2, During-feed 2, and Post-feed 2). In this way, we created a time-dependent dominant frequency distribution and partition map for each sub-feeding period, as demonstrated in Fig. 8a. Each red box represents the dominant frequency band during each minute, and only one box appears per minute.

##### Step 4

The total number of red boxes in each GR band shown in Fig. 8a represents the total time, T_GR_, during which the corresponding GR was dominant for the selected time period, such as pre-, during, or post-feed (within Feeding 1 or Feeding 2). Only one T_GR_ was chosen per minute among bradygastria, normogastria, and tachygastria.

##### Step 5

The percentage of time spent in each GR band during a selected time period (such as Pre-feed 1, During-feed 1, Post-feed 1, Pre-feed 2, During-feed 2, and Post-feed 2) was quantified by dividing T_GR_ ( = number of red boxes in the respective frequency band) by the total minutes during the chosen feeding period, as outlined in Fig. 8b. Using this method, we obtained respective % bradygastria, % normogastria, and % tachygastria for each neonate in all sub-feeding periods.

##### Step 6

Then we averaged % bradygastria, % normogastria, and % tachygastria, respectively, over two Feeding periods for each sub-feeding period of each neonate (i.e., Pre-feed 1, Pre-feed 2; During-feed 1, During-feed 2; Post-feed 1, Post-feed 2). This was done to examine whether the percentage time spent on each GR band depended on feeding conditions.

##### Step 7

The percentage time spent in each GR frequency band during each sub-feeding period was plotted against the GA of each neonate. Linear regression was performed to determine the linear relationship between GA versus percentage time spent at each GR band. The significance level was set to be *p*<0.05.

## Supplementary Information

Supplementary Information.

## Data Availability

The datasets generated during and/or analysed during the current study are available from the corresponding author on reasonable request.
