# Supplementary material for: Frequency-specific electrogastrography as a non-invasive tool to measure gastrointestinal maturity in preterm infants
Source: Sci Rep. 2022 Dec 1;12:20728. doi: 10.1038/s41598-022-24110-y (PMC9715709; doi:10.1038/s41598-022-24110-y)
Supplement: Supplementary file 1 — Supplementary Information. [file 41598_2022_24110_MOESM1_ESM.docx]

**Frequency-Specific Electrogastrography as a Non-Invasive Tool**

**to Measure Gastrointestinal Maturity in Preterm Infants**

**Akhil Chaudhari^1^, Xinlong Wang^1^, Lindsay Roblyer^2^, Rinarani Sanghavi^3^, Hanli Liu^1^,**

**and Eric B. Ortigoza^2^***

**Supplementary Information**

**Supplementary Table S1.** **Demographics**

|  | (N=51) |
| --- | --- |
| **Gestational age at birth** |  |
| Early (23-28 weeks) | 25 (49) |
| Mid (29-33 weeks) | 22 (43) |
| Term (≧37 weeks) | 4 (8) |
| **Birth weight, kg** | 1.23 (0.67-4.25) |
| **Sex** |  |
| Female | 26 (51) |
| Male | 25 (49) |
| **Race/Ethnicity** |  |
| Black Non-Hispanic | 14 (27) |
| White Non-Hispanic | 1 (2) |
| Hispanic/Latino | 35 (69) |
| Asian | 1 (2) |

Median (Range) for continuous variables and n (%) for categorical.

**Supplementary Table S2.** **List of p-values for comparisons among different pairs of *m*PSD values at three GR bands during three feeding periods.**

|  |  | **Pre-feed** | **During-feed** | **Post-feed** |
| --- | --- | --- | --- | --- |
| **Bradygastria** | Early vs. Mid GA | 0.43 | 0.62 | 0.11 |
|  | Mid GA vs. Term | 0.13 | 0.04* | 0.49 |
|  | Early GA vs. Term | 0.2 | 0.01* | 0.85 |
| **Normogastria** | Early vs. Mid GA | 0.11 | 0.01* | 0.02* |
|  | Mid GA vs. Term | 0.21 | 0.04* | 0.42 |
|  | Early GA vs. Term | 0.22 | 3.5x10^-5*^ | 0.61 |
| **Tachygastria** | Early vs. Mid GA | 0.01* | 0.004* | 0.006* |
|  | Mid GA vs. Term | 0.03* | 0.32 | 0.36 |
|  | Early GA vs. Term | 0.08 | 2.4x10^-4*^ | 0.46 |

Note: The p values were derived by one-way ANOVA followed by Tukey’s test. *significance level with *p* < 0.05.

**Supplementary Table S3. Feed Information For Each Group**

|  | **Early (n=25)** | **Mid (n=22)** | **Term (n=4)** | ***p*-value** |
| --- | --- | --- | --- | --- |
| **Formula** | 5 (20) | 10 (45) | 4 (100) | 0.37 |
| **Maternal Own Milk (MOM)** | 12 (48) | 10 (45) | 0 (0) | 0.37 |
| No Human Milk Fortifier | 1 (4) | 0 (0) | 0 (0) |  |
| Human Milk Fortifier | 11 (44) | 10 (45) | 0 (0) |  |
| **Donor Human Milk (DHM)** | 1 (4) | 0 (0) | 0 (0) | 0.37 |
| No Human Milk Fortifier | 0 (0) | 0 (0) | 0 (0) |  |
| Human Milk Fortifier | 1 (4) | 0 (0) | 0 (0) |  |
| **Mixture of MOM and Formula** | 7 (28) | 2 (9) | 0 (0) | 0.37 |
| **Average Calories (Kcal/oz)** | 24.0 (20-30) | 24.0 (20-27) | 19.0 (19-19) | 0.37 |
| **Average Volume (mL/Kg)** | 18.3 (6.5-22.1) | 19.3 (5.2-28.8) | 14.3 (12.1-17.7) | 0.37 |

Note: P-values derived by Kruskal Wallis Test. Kilocalories per ounce (Kcal/oz), milliliter per kilogram (mL/Kg).

**Supplementary Table S4. Similarities and Differences Between Current Study and**

**Previous Studies^1-4^ in pre-term and term infants**

| **Category** | **Similarities** | **Differences in our approach** |
| --- | --- | --- |
| EGG recording | EGG taken in pre- and post-feed periods | EGG taken inclusive of *during-feed* period |
| Data analysis methods | Frequency-domain analysis (FD) | We used direct and simple PSD data analysis |
| Quantified parameters | Derived from FD analysis of EGG time series | We quantified mean power spectral density (mPSD) at 3 gastric waves instead of conventional EGG analysis parameters (e.g., dominant frequency, dominant power, power ratio). |
| Method to decide the time spent in each gastric band | Determination of the percent of time spent in each of the three gastric rhythm bands | We utilized continuous wavelet transform (CWT) for non-stationary data analysis to calculate the percent of time spent in each gastric rhythm. |
| Major results /observations | Identification in parameter differences among groups or feeding periods of neonates | We reported linear and significant correlation and dependence between the increasing gestational age and percentage time spent in different gastric rhythms. Results from other groups are described below.** |

**

Lange et. al.^1^ did not detect any significant difference between feeding periods or groups of neonates based on conventional EGG analysis parameters. Tomomasa et al.^2^ did not identify significant difference in mean amplitude of EGG waves. Also, they concluded that EGG of neonates is different from that of adults and can be sensitive to the site positions of EGG electrodes. Precioso et al.^3^ did not observe significant difference between preterm and full-term neonates during the pre- and post-feeding periods. They reported that gastric myoelectrical activity in pre-term and full-term neonates is immature, as compared with older neonates, children and adults. Patterson et al. ^4^ showed that as neonates aged from birth to age of 2 years, the mean percentage recorded time of normogastria increased significantly while those of tachygastria and bradygastria reduced. The pattern of EGG in the normal neonates differs from that in adults. Tachygastria and bradygastria are seen more often in neonates, with fewer periods in normogastria. An increase in the percentage in the latter is expected over time.

**References:**

1 Lange, A., Huang, L. & Funch-Jensen, P. Electrogastrography in neonates. *Neurogastroenterol Motil* **17**, 512-517, doi:10.1111/j.1365-2982.2005.00656.x (2005).

2 Tomomasa, T., Miyazaki, M., Nako, Y. & Kuroume, T. Electrogastrography in neonates. *J Perinatol* **14**, 417-421 (1994).

3 Precioso, A. R., Pereira, G. R. & Vaz, F. A. Gastric myoelectrical activity in neonates of different gestational ages by means of electrogastrography. *Rev Hosp Clin Fac Med Sao Paulo* **58**, 81-90, doi:10.1590/s0041-87812003000200005 (2003).

4 Patterson, M., Rintala, R. & Lloyd, D. A. A longitudinal study of electrogastrography in normal neonates. *J Pediatr Surg* **35**, 59-61, doi:10.1016/s0022-3468(00)80014-7 (2000).
